# Identification of Chemokines Associated with the Recruitment of Decidual Leukocytes in Human Labour: Potential Novel Targets for Preterm Labour

**DOI:** 10.1371/journal.pone.0056946

**Published:** 2013-02-22

**Authors:** Sarah A. Hamilton, Clare L. Tower, Rebecca L. Jones

**Affiliations:** Maternal and Fetal Health Research Centre, Institute of Human Development, University of Manchester, Manchester Academic Health Sciences Centre, Manchester, United Kingdom; Otto-von-Guericke University Magdeburg, Germany

## Abstract

Current therapies for preterm labour (PTL) focus on arresting myometrial contractions but are largely ineffective, thus alternative therapeutic targets need to be identified. Leukocytes infiltrate the uterus around the time of labour, and are in particularly abundant in decidua (maternal-fetal interface). Moreover, decidual inflammation precedes labour in rat pregnancies and thus may contribute to initiation of labour. We hypothesized that chemokines mediate decidual leukocyte trafficking during preterm labour (PTL) and term labour (TL), thus representing potential targets for preventing PTL. Women were recruited into 4 groups: TL, term not in labour (TNL), idiopathic PTL and PTL with infection (PTLI). Choriodecidual RNA was subjected to a pathway-specific PCR array for chemokines. Differential expression of 12 candidate chemokines was validated by real time RT-PCR and Bioplex assay, with immunohistochemistry to confirm cellular origin. 25 chemokines were upregulated in choriodecidua from TL compared to TNL. A similar pattern was detected in PTL, however a distinct profile was observed in PTLI consistent with differences in leukocyte infiltration. Upregulation of CCL2, CCL4, CCL5, CXCL8 and CXCL10 mRNA and protein was confirmed in TL, with CCL8 upregulated in PTL. Significant correlations were detected between these chemokines and decidual leukocyte abundance previously assessed by immunohistochemical and image analysis. Chemokines were primarily expressed by decidual stromal cells. In addition, CXCL8 and CCL5 were significantly elevated in maternal plasma during labour, suggesting chemokines contribute to peripheral inflammatory events during labour. Differences in chemokine expression patterns between TL and idiopathic PTL may be attributable to suppression of chemokine expression by betamethasone administered to women in PTL; this was supported by in vitro evidence of chemokine downregulation by clinically relevant concentrations of the steroid. The current study provides compelling evidence that chemokines regulate decidual leukocyte recruitment during labour. The 6 chemokines identified represent potential novel therapeutic targets to block PTL.

## Introduction

Preterm birth contributes to substantial neuro-cognitive, pulmonary, and ophthalmologic morbidity and accounts for around 25% of neonatal deaths [1]. Preterm birth rates in the UK and USA are 7.3% and 12.8% respectively [2] and continue to rise despite advancing knowledge of risk factors for preterm labour (PTL) and the introduction of public health and medical interventions [3], [4], [5]. Prolonging a pregnancy from 30 to 34 weeks gestation decreases neonatal mortality from 9.6% to 0.9% [6], however, tocolytics currently in use have limited effectiveness and on average only delay labour by up to 48 hours [7]. Understanding more about the mechanisms of labour is essential to identify targets for novel and more effective therapies to stop or prevent PTL.

Inflammatory processes have been associated with preterm (both idiopathic and infection-associated) and term labour [8]. Leukocyte infiltration and upregulation of cytokines/chemokines occurs in cervix, fetal membranes and myometrium during labour at term [9], [10]; these inflammatory processes interact with stretch and endocrine signals to initiate and amplify the labour cascade. The involvement of the decidua, (endometrium of pregnancy) in parturition has been largely overlooked, despite it being a highly immunologically active tissue and ideally placed at the maternal-fetal interface to coordinate inflammatory processes during labour [10], [11]. We recently demonstrated leukocyte infiltration of the decidua occurs during labour, both at term and preterm in human pregnancies [12]. Macrophages were the predominant leukocyte subtype present in term and idiopathic PTL; an influx of these cells could activate parturition processes in decidua and neighbouring maternal and fetal tissues through their release of matrix metalloproteases, cytokines and prostaglandins [13], [14]. Distinct decidual leukocyte infiltration patterns between idiopathic and infection associated PTL confirmed differing aetiologies, with the latter characterised with massive neutrophil influx [12].

Using a rat model, we also detected a significant increase in macrophage infiltration of the decidua in the days prior to labour, which preceded inflammatory changes in the myometrium [12]. This suggests that decidual inflammatory events participate in the initiation, rather than occurring as a consequence, of labour thus consolidating the hypothesis first proposed in the 1980s that decidual activation is an early event in the labour cascade [15]. Therefore, decidual inflammatory processes represent a potential therapeutic target for the prevention of PTL.

Studies by Gomez-Lopez et al have demonstrated potent chemotactic activity of fetal membrane extracts, particularly towards macrophages and neutrophils, following term labour [16]; however the chemotactic agents responsible have not been identified. Chemokines are likely candidates due to their chemotactic properties for leukocytes and their instrumental role in instigating and coordinating inflammatory reactions. Previous studies have shown altered chemokine mRNA expression by fetal membranes, myometrium and cervix with labour [17], [18], [19], [20], [21], [22]. However, approximately 50 chemokines exist and previous studies have generally focussed on a few selected chemokines and decidual chemokine expression has been largely neglected. Around 50 chemokine ligands exist, with such redundancy essential to defend against foreign infectious agents. Thus it is unlikely that a single chemokine will be instrumental for mediating the critical process of labour, reinforcing the need for an array rather than candidate gene approach. The current study employed an unbiased gene array strategy to identify the key chemokines upregulated by the decidua during term and PTL, combined with detailed analysis of chemokine protein levels. Chemokine levels in peripheral blood samples from women in term and PTL were also analysed as putative mediators of peripheral leukocyte activation and systemic inflammatory processes reported during labour [23].

We hypothesised that a distinct chemokine profile in the decidua is responsible for regulating leukocyte infiltration during labour at term and preterm. Our aim was to identify the specific chemokines involved in term and PTL, which may be potential targets for blocking PTL.

## Methods

### Clinical Study Groups

Pregnant women who delivered at term (37–42 weeks) and in PTL (24–35 weeks) were recruited from St Mary’s Hospital in Manchester with informed written consent as previously described [12]. This study was approved by the North West Local Research Ethics Committee (#08/H1010/55). Strict inclusion and exclusion criteria were used to recruit women into 4 study groups for blood sampling and tissue collection: term not in labour (TNL, elective Caesarean section at term without labour, n = 14), normal term labour (TL, n = 14), idiopathic PTL (PTL, n = 10) and PTL with infection (PTLI, n = 10). A further cohort of pregnant women at 28 weeks gestation attending antenatal clinics with no signs of PTL was recruited for blood samples only (PTNL, n = 10). Women with PTLI were defined as those with 2 or more of the following infection criteria: spontaneous rupture of membranes (SROM) >48 hours before labour, temperature: >37.6°C, white cell count >15, C-reactive protein >10, positive high vaginal swab. All fetal membranes from pregnancies in this group exhibited histological features of chorioamnionitis. Women in the idiopathic PTL group had no signs of infection using criteria detailed above and fetal membranes exhibited no histological features of chorioamnionitis. There were no identifiable pathological causes of PTL (maternal or fetal). Women in all study groups delivered infants of normal birth weight (defined as an individualised birth weight centile between 10–90) [24]. Exclusion criteria for all groups were maternal hypertension, proteinuria, diabetes and fetal anomalies. Demographic and biophysical data was obtained, together with detailed obstetric history. A further 7 patients undergoing elective Caesarean section at TNL were recruited for in vitro studies of choriodecidua.

Blood samples were obtained from women in TL and PTL when in established labour (>3 cm cervical dilatation and regular contractions). Blood samples from the women in TNL and PTNL groups were obtained prior to elective Caesarean delivery or at routine antenatal visits respectively. Fetal membranes were collected within 30 minutes of delivery. Choriodecidual tissue was sampled from the midzone area, distant from the rupture side and placenta and either snap frozen or placed in RNA later (Sigma, Poole, UK) prior to freezing at −80°C. Intact midzone fetal membranes (0.5 cm×2 cm) were tightly rolled, fixed in 10% neutral buffered formalin, and processed into paraffin blocks, orientated such that transverse cross sections were cut. For in vitro studies, choriodecidua was sampled.

Sample sizes were determined based on existing studies in the literature describing chemokine/cytokine expression by gestational tissues in term labour [10], [21], [25], [26]. Power calculations determined that sample sizes of 5–14 per group (depending on the cytokine) would be required to detect similar differences in mean cytokine expression with a power of 80% at the 5% level of significance.

### PCR Arrays for Chemokines and Receptor

Total RNA was extracted from RNA later treated choriodecidual tissue from each of the four study groups using TRIZOL (Sigma), followed by clean up through RNeasy columns (Qiagen, Crawley, UK) and DNase treatment using DNA-free kit (Invitrogen). RNA quality and quantity was assessed by UV spectroscopy (Eppendorf, Stevenage, UK) and Ribogreen analysis (Life Technologies, Paisley, UK). RNA samples were pooled to create 4 study group pools and 1000 ng of pooled RNA was converted to cDNA using the RT^2^First Strand Kit (SABiosciences, Frederick, USA). cDNA was subsequently combined with the RT^2^qPCR Master Mix according to the manufacturer’s instructions and applied to Pathway specific RT^2^ Profiler PCR Arrays (SABiosciences) for chemokines and chemokine receptors. This included a total of 96 chemokines, chemokine receptors, cytokines, inflammatory associated genes and control genes. A MX3000 thermal cycler (Agilent, Berkshire, UK) was used with the following cycle conditions: 1 cycle of 95°C for 10 min, 40 cycles of: 95°C for 15s and 60°C for 1 min. During each annealing phase SYBR green fluorescence was detected and recorded. The cycle threshold (Ct) values were recorded for each of the 96 wells and expression levels were calculated using a standard equation (2^(−Ct)^). All expression levels were normalised to housekeeping gene RPL13 which did not vary significantly between the study groups. The effect of labour on chemokine expression was examined by calculating the fold change in expression levels in the TL, PTL and PTLI groups compared with TNL.

### Quantitative RT-PCR

To validate the results from the PCR arrays, quantitative PCR for selected chemokines was performed on the individual samples which contributed to the original RNA pools. RNA samples (250 ng) were reverse transcribed using Stratagene AffinityScript Multi-Temperature cDNA Synthesis kit (Agilent). A standard curve of reference cDNA (spleen or placenta, Agilent) was generated by serial dilution (x-5). The cDNA samples were diluted between 1∶10–1∶50 depending on the chemokine being analysed. cDNA samples were added to a PCR reaction master mix containing: SYBR Brilliant II Green Master Mix (Agilent), 0.25 µM primers, and 2 µM of Reference Dye (ROX) in RNase-free PCR strip tubes (Sarstedt, Leicester, UK). All reactions were performed in duplicate. PCRs were performed in a MX3000P (Agilent) using the following cycle conditions: 1 cycle of 95°C for 10 min, 40 cycles of: 95°C for 30s, X°C for 1 min (annealing temperature, where X is dependent on the primer set, see Table 1), and 72°C for 1 min. The expression level of chemokine in each sample was extrapolated from the standard curve, and normalised to RPL13.

**Table 1:** Primers used for real time PCR analyses.

Primer Set	Sequence	Anneal Temp (°C)	Reference
**CCL2**	AATCAATGCCCCAGTCACCTGC CGGAGTTTGGGTTTGCTTGTCC	60	[54]
**CCL3**	GTCATCTTCCTAACCAAGCG TGTGGCTGTTTGGCAACAAC	60	[55]
**CCL4**	AGGAAGCTTCCTCGCAACTT AGTCCTGAGTATGGAGGAGA	60	[56]
**CCL5**	ACCAGTGGCAAGTGCTCCA TCCCGAACCCATTTCTTCTCT	60	[55]
**CCL8**	GAGAGCTACACAAGAATCACCAA TGGTCCAGATGCTTCATGGAA	60	PrimerBank ID 22538816a1
**CCL18**	CCTGCAGCTGCCCTCCTTGTC CACTTCTTATTGGGGTCAGC	60	[57]
**CXCL1**	ATAGCCACACTCAAGAATG TCTGCAGCTGTGTCTCTCTT	60	[58]
**CXCL6**	GCAGTTACCAATCGTTTTGGGG AGAGCTGCGTTGCACTTGTT	60	PrimerBank ID 4506851a1
**CXCL8**	CACCGGAAGGAACCATCTCACT TCAGCCCTCTTCAAAAACTTCTCC	59	[41]
**CXCL9**	CCAGTAGTGAGAAAGGGTCGC TGGGGAAATTGTTTAAGCTCTT	60	PrimerBank ID 4505187a1
**CXCL10**	TGAAATTATTCCTGCAAGCCAA GACATCTCTTCTCACCCTTCTTT	60	PrimerBank ID 4504701a1
**CX3CL1**	GCTTTGCTCATCCACTATCAACA GCTCCAGGCTACTGCTTTCG	57	[41]
**RPL13**	CGAGGTTGGCTGGAAGTACC CTTCTCGGCCTGTTTCCGGTAG	59	PrimerBank ID 6912634a1

### Bioplex Pro Cytokine Chemokine Assay and ELISAs

Protein lysates were generated from snap frozen midzone choriodecidual tissue from the 4 patient study groups using Bio-plex Cell Lysis Kit (BioRad, Hemel Hempstead, UK). A Biorad protein assay was performed to determine protein concentrations. Bioplex Pro Cytokine Chemokine Assays (BioRad) were performed as per manufacturer’s guidelines using 5 µg protein lysate per well. Chemokines examined by this method were CCL2, CCL3, CCL4, CCL5, CXCL1, CXCL8 and CXCL10. Maternal plasma samples from women in labour at term and preterm (idiopathic PTL), together with non-labouring controls at term and 28 weeks were included in the assay. ELISAs (RayBiotech, Norcross GA, USA) were performed to analyse protein expression of CCL8 and CXCL9 in choriodecidual lysates. Assays were performed according to the manufacturer’s instructions using 5 µg protein lysate per well. For both methods chemokine concentrations (pg/ml) were extrapolated from the standard curve.

### Immunohistochemistry on Fetal Membranes

Immunohistochemistry was performed to identify the cellular layer (chorion vs. decidua) and specific cell types responsible for chemokine production, as previously described [27]. Primary antibodies against chemokines (CCL2 2 µg/ml, CCL4 1 µg/ml, CCL5 4 µg/ml, CCL8 2 µg/ml, CXCL8 2 µg/ml, CXCL10 2 µg/ml; Santa Cruz, Heidelberg, Germany) were applied to tissue sections overnight at 4°C. Negative controls were performed by pre-absorption of each antibody with a 10-fold excess of the relevant chemokine peptide (Santa Cruz). Biotinylated rabbit anti-goat IgG (2.6 µg/ml, Dako) was applied followed by detection with avidin-peroxidase (5 µg/ml, Sigma) and DAB (Sigma). Stained fetal membranes were examined to identify the cellular source of each chemokine, and photographed using an Olympus microscope and QI-Cam digital camera with Image-Pro Plus 7.0 (Media Cybernetics, Marlow, UK).

### Treatment of Choriodecidual Explants with Betamethasone

To determine whether betamethasone administered to women in PTL to improve neonatal outcomes altered chemokine expression, choriodecidual explants (2–3 mm^2^) from term elective Caesarean sections (n = 7) were treated for 24 hours with betamethasone at concentrations spanning those in maternal blood following antenatal administration treatment (100 nM, 500 nM, 1000 nM) [28] or vehicle (0.01% ethanol) in serum-free DMEM/F12 (Invitrogen) supplemented with 1% penicillin-streptomycin-glutamine (Sigma). RNA was extracted and Q-PCR was performed as above for the selected candidate genes. The PCR data were normalised to RPL13 and presented as fold change from vehicle control.

### Statistical Analysis

Kruskall Wallis test with Dunn’s multiple comparison post hoc test was used to detect statistically significant differences in chemokine mRNA and protein expression between the study groups (TNL, TL, PTL and PTLI). The chemokine expression data obtained were correlated with our previously published data of leukocyte subpopulations present in the decidua (obtained by detailed immunohistochemical and image analysis studies using the same tissue samples) [12]. Data were combined for all study groups (with data from PTLI excluded for some analyses as described in the results), to determine overall relationships between the level of expression of each chemokine by the decidua (at both the mRNA and protein level) and the number of each leukocyte subtype (total CD45^+^ leukocytes, CD68^+^ macrophages, neutrophils, CD3^+^ T cells and NK cells) present in the decidua. Spearman’s rank correlation was performed to identify significant relationships. Wilcoxon Signed Rank Test was used to compare expression levels in betamethasone treated explants compared to controls.

## Results

### Patient Demographics

As previously reported, there were no significant differences in demographic or biophysical characteristics between the patient groups [12], except for a significant difference in gestational age at delivery for both PTL groups compared to term groups (p<0.001), and a higher rate of previous PTL, late miscarriage and cervical surgery in the PTL groups (p<0.05 for all).

### Choriodecidual Chemokine Expression Profiles in Labour

Chemokine and receptor pathway specific PCR arrays were performed to identify changes in chemokine expression profiles in choriodecidua with labour. Individual comparisons were made between the 3 labour groups (TL, PTL, PTLI) and the non-labour group (TNL). 69 genes were upregulated and 1 gene downregulated ≥2-fold in choriodecidua in the TL compared to the TNL group (Table S1). Twenty six of those genes upregulated with labour at term were chemokines (14 CC chemokines, 10 CXC chemokines, XCL1 and CX3CL1; Table 2). In the PTL group, 53 genes were upregulated and 4 downregulated compared to TNL (Table S1); of those upregulated, 19 were chemokines and of those downregulated 2 were chemokines (Table 2). The 19 chemokines upregulated in PTL were also upregulated in the TL group, although the degree of upregulation was generally lower than in TL, with the exception of CCL8. The downregulated chemokines were CCL13 and CCL16. The PTLI group exhibited a markedly distinct inflammatory gene profile, with 31 genes upregulated and 24 downregulated compared to TNL (Table S1). Fifteen chemokines were upregulated in PTLI compared with TNL (Table 2). There were considerable differences in the chemokine profile between the PTL and PTLI groups, most notably a downregulation of CC chemokines, whilst CXC chemokines were highly upregulated (2–143 fold, Table 2). Of the upregulated chemokines in common with the other labour groups, CCL2 and 3 and CXCL1, 5, 8 were expressed at higher levels in PTLI than in TL or PTL.

**Table 2:** Chemokine mRNA profiles during term and preterm labour.

Chemokines	TL vs. TNL Fold change	PTL vs. TNL Fold change	PTLI vs. TNL Fold change
***CC Chemokines***			
CCL1	5.2	1.4	−10.9
**CCL2**	**3.9**	**3.0**	**11.5**
**CCL3**	**2.4**	**1.7**	**34.9**
**CCL4**	**13.2**	**7.5**	**−16.2**
**CCL5**	**4.4**	**2.8**	**1.3**
CCL7	U	U	U
**CCL8**	**25.9**	**7.3**	**1.8**
CCL11	45.1	9.4	D
CCL13	12.5	D	−1.5
CCL15	U	U	–
CCL16	9.9	−14.4	D
CCL17	13.9	2.8	2.6
**CCL18**	**16.3**	**27.0**	**−1.5**
CCL19	2.7	−1.8	−2.3
***CXC chemokines***			
**CXCL1**	**35.1**	**27.2**	**59.1**
CXCL2	119.8	42.6	24.9
CXCL3	U	U	U
CXCL5	18.4	16.7	58.3
**CXCL6**	**U**	**U**	**U**
**CXCL8**	**29.9**	**27.0**	**144.6**
**CXCL9**	**5.3**	**3.9**	**2.6**
**CXCL10**	**17.4**	**14.9**	**10.0**
CXCL11	44.5	17.9	7.0
CXCL12	1.4	1.9	2.1
CXCL13	2.3	1.6	2.2
***Other chemokines***			
XCL1	3.8	1.1	−19.0
**CX3CL1**	**3.5**	**2.5**	**−1.3**

### Gene Array Validation Studies

**Figure 1:**
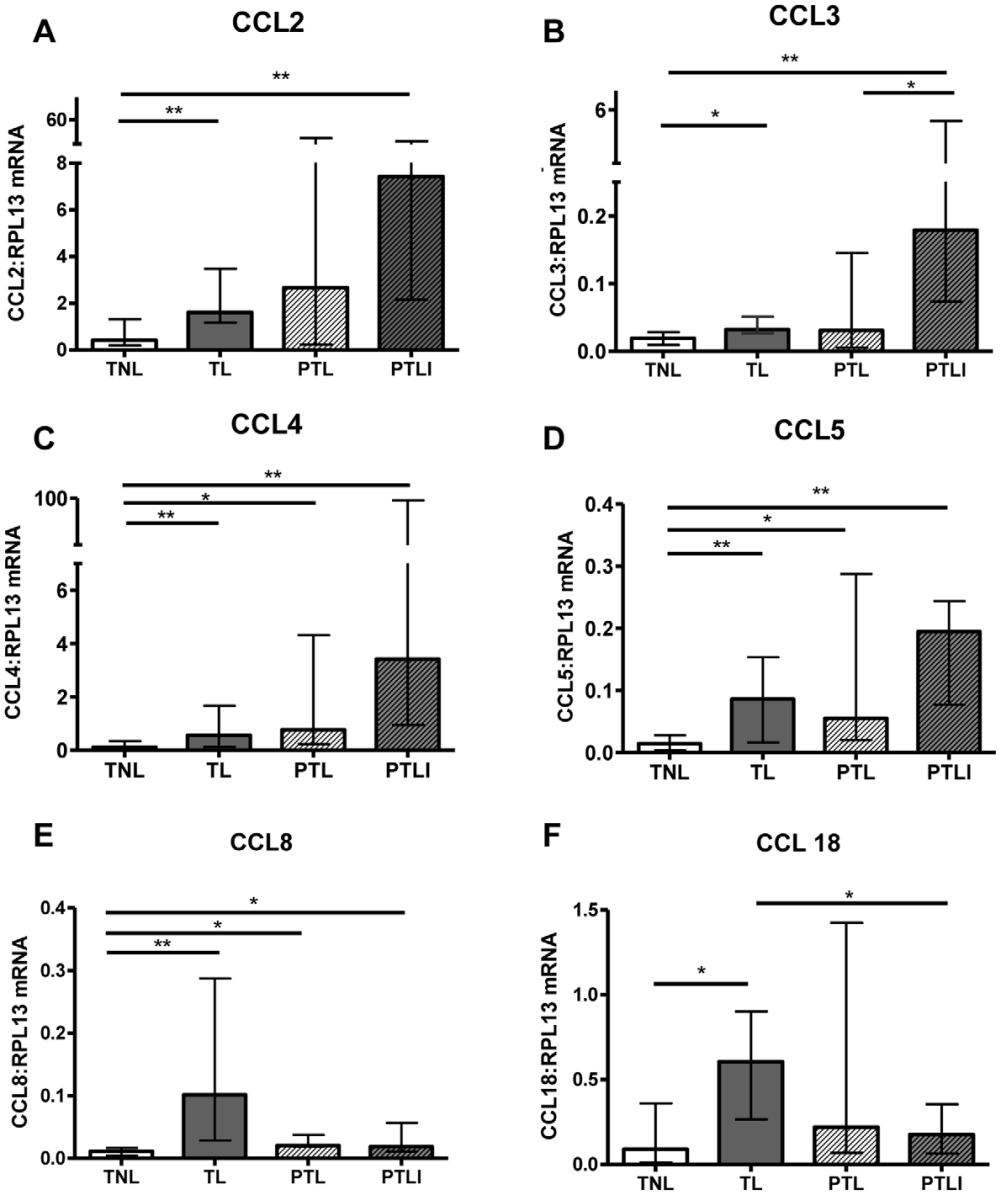
Choriodecidual CC chemokine mRNA expression in the four study groups. Chemokine mRNA expression was normalised to RPL13 mRNA expression levels. (A) CCL2, (B) CCL3, (C) CCL4, (D) CCL5, (E) CCL8, (F) CCL18. Individual replicates and the median are shown, n = 10–14/study group. TNL = Term not in labour, TL = Term labour, PTL = Preterm labour and PTLI = Preterm labour with infection. Data are median and interquartile range, *p<0.05 **p<0.01 (Kruskall-Wallis with Dunn’s post hoc test).

From the PCR arrays 12 candidate chemokines were chosen for more detailed analysis: CCL2, CCL3, CCL4, CCL5, CCL8, CCL18, CXCL1, CXCL6, CXCL8, CXCL9, CXCL10 and CX3CL1, based on their level of fold change in the TL and PTL groups compared to the TNL group, their expression level within the tissue and their chemoattractant properties. To validate the PCR array findings for each of the candidate genes QPCR was performed on the individual RNA samples from the 4 study groups (n = 10–14/group). RPL13 was used as a housekeeping gene as its expression did not differ between groups. The upregulation of CC chemokine mRNA expression (CCL2, CCL3, CCL4, CCL5, CCL8, CCL18) with TL compared to TNL was successfully validated by PCR (Fig. 1, p<0.05 or 0.01). Of these, only CCL4, CCL5 and CCL8 were significantly upregulated at the mRNA level in choriodecidua from PTL compared to TNL (Fig. 1, p<0.05).

**Figure 2:**
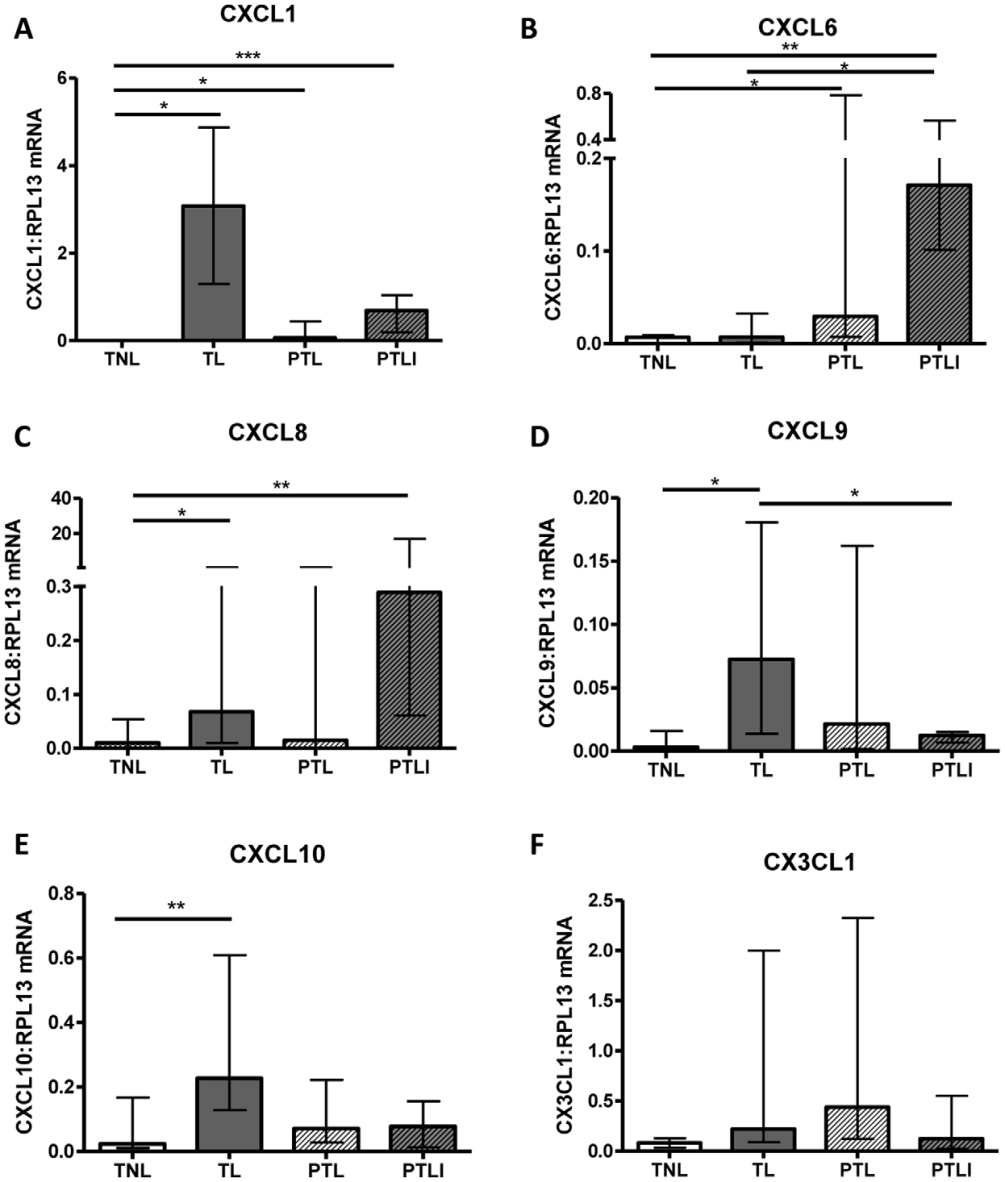
Choriodecidual CXC and CX3C chemokine mRNA expression in the four study groups. Chemokine mRNA expression was normalised to RPL13 mRNA expression levels. (A) CXCL1, (B) CXCL6, (C) CXCL8, (D) CXCL9, (E) CXCL10, (F) CX3CL1. Individual replicates and the median are shown, n = 10–14/study group. TNL = Term not in labour, TIL- Term labour, PTL = Preterm labour and PTLI = Preterm labour with infection. Data are median and interquartile range, *p<0.05 **p<0.01, ***p<0.001 (Kruskall-Wallis with Dunn’s post hoc test).

Five CXC chemokines were studied; of these CXCL1, CXCL8, CXCL9 and CXCL10 were significantly elevated at the mRNA level in TL vs. TNL (Fig. 2, p<0.05 or 0.01). Only CXCL1 and CXCL6 were significantly upregulated in PTL (p<0.05). A trend for upregulation of CX3CL1 was detected in PTL compared to TNL which narrowly failed to reach significance (Fig. 2, p = 0.07).

A number of chemokines were upregulated in PTLI compared to TNL: CCL2, CCL3, CCL4, CCL5, CCL8, CXCL1, CXCL6 and CXCL8 (p<0.05, 0.01 or 0.001; Fig. 1 and 2). In addition, significantly higher expression of CCL3 was detected in the PTLI group compared to PTL (p<0.05), with a similar trend for CXCL8 (p = 0.07).

### Choriodecidual Chemokine Protein Expression

**Figure 3:**
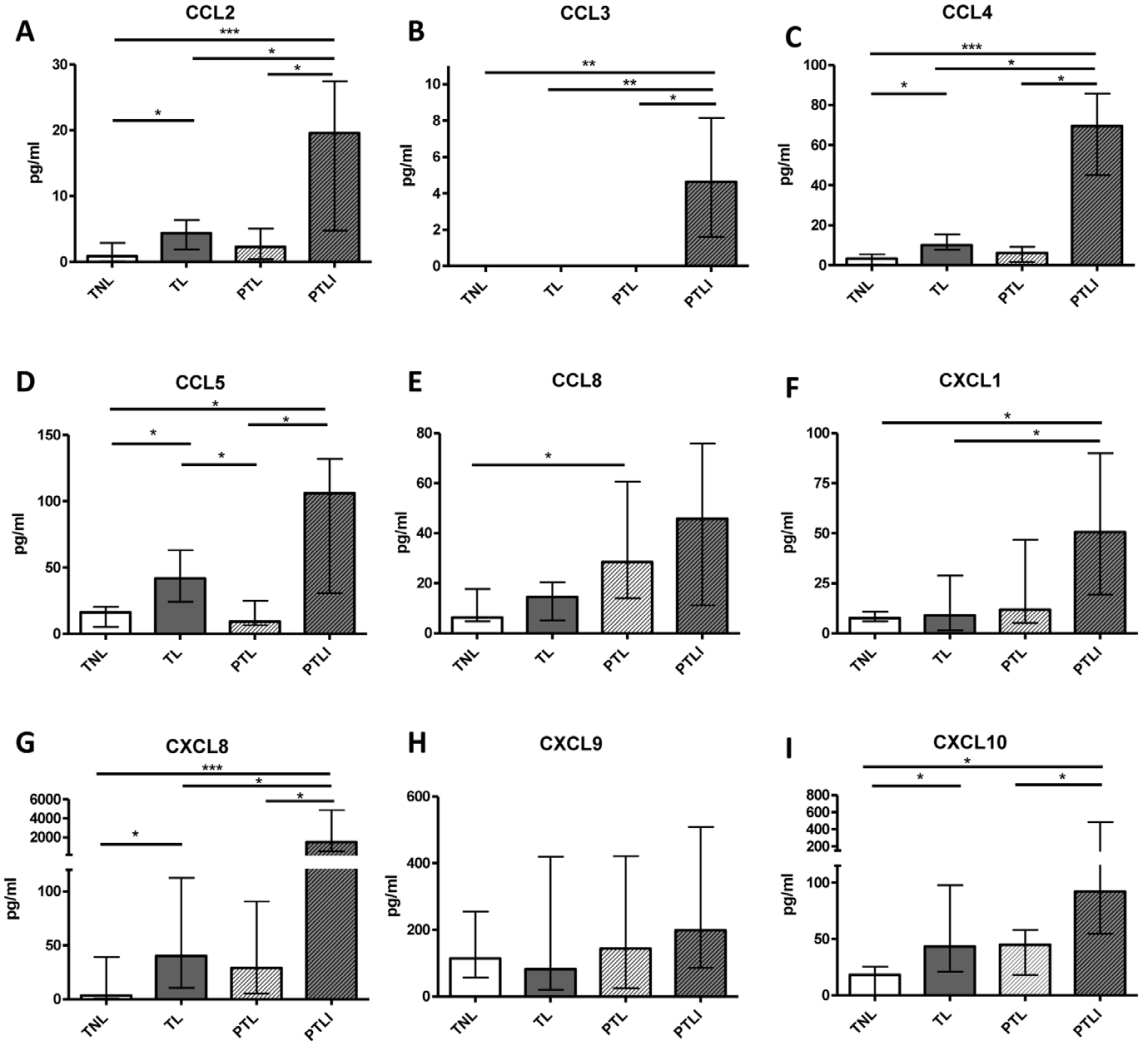
Concentrations of chemokine protein (pg/ml) in choriodecidual tissue lysates in the four study groups. (A) CCL2, (B) CCL3, (C) CCL4, (D) CCL5, (E) CCL8, (F) CXCL1, (G) CXCL8, (H) CXCL9, (I) CXCL10. Individual replicates and the median are shown, n = 10/study group. TNL = Term not in labour, TL = Term labour, PTL = Preterm labour and PTLI = Preterm labour with infection. Data are median and interquartile range, *p<0.05, **p<0.01, ***p<0.001 (Kruskall-Wallis with Dunn’s post hoc test).

Bio-plex Pro Assays or ELISAs were performed on choriodecidual lysates to quantify the protein concentrations of the 9 chemokines (CCL2, CCL3, CCL4, CCL5, CCL8, CXCL1, CXCL8, CXCL9, CXCL10) that were significantly altered by TL or PTL at the mRNA level. Protein concentrations of CC chemokines: CCL2, CCL4, CCL5, and CXC chemokines: CXCL8 and CXCL10, were significantly increased in choriodecidual lysates from TL compared with TNL (Fig. 3, p<0.05). The only changes in chemokine protein levels in PTL were higher concentrations of CCL8 compared to TNL (p<0.05) and lower concentrations of CCL5 than TL (p<0.05). All of the chemokines examined, except CXCL9 and CCL8, had significantly increased protein levels in the PTLI group when compared with TNL. CCL2, CCL3, CCL4, CXCL8 were also more abundant in PTLI than TL and PTL, whilst CCL5, CCL7 and CXCL10 were more abundant in PTLI than PTL only.

### Summary of Chemokine mRNA Expression and Protein Expression

The data showing mRNA expression and protein expression of the candidate chemokines are collated in Table 3. CCL2, CCL4, CCL5, CXCL8, and CXCL10 were consistently upregulated in choriodecidua at both the mRNA and protein expression with TL compared to TNL. CCL8 was the only chemokine that was significantly upregulated both at the mRNA and protein level in choriodecidua from PTL compared to TNL.

**Table 3:** Summary of changes in chemokine mRNA and protein expression in term and preterm labour.

Chemokine	TL vs. TNL		PTL vs. TNL	
	mRNA	Protein	mRNA	Protein
**CCL2**	**Upregulated** **	**Upregulated** *	NC	NC
**CCL3**	**Upregulated** *	NC	NC	NC
**CCL4**	**Upregulated** **	**Upregulated** *	**Upregulated** *	NC
**CCL5**	**Upregulated** **	**Upregulated** *	**Upregulated** *	NC
**CCL8**	**Upregulated** **	NC	**Upregulated** *	**Upregulated** *
**CCL18**	**Upregulated** *	**–**	NC	–
**CXCL1**	**Upregulated** *	NC	**Upregulated** *	NC
**CXCL6**	NC	–	**Upregulated** *	–
**CXCL8**	**Upregulated** *	**Upregulated** *	NC	NC
**CXCL9**	**Upregulated** *	NC	NC	NC
**CXCL10**	**Upregulated** **	**Upregulated** *	NC	NC
**CX3CL1**	NC	–	NS upregulation (p = 0.07)	–

TL = term labour, TNL = term not in labour, PTL = preterm labour, NC = no change, – = not examined.

* p<0.05,

** p<0.01.

### Correlation Analyses

Correlations using Spearman Rank test were performed between the expression level of these 6 chemokines and numbers of leukocyte subtypes present in the decidua, to support potential functional roles in mediating leukocyte recruitment. PTLI was excluded from the analysis as the production of chemokines by the massive neutrophil component skewed the results. There were no correlations with chemokine mRNA expression and total leukocyte numbers (CD45^+^), however CCL5 and CXCL10 mRNA expression positively correlated with macrophage (CD68^+^) numbers (p<0.05), with trends for correlations between CCL4, CCL8 and CXCL1 narrowly failing to reach significance (p = 0.06/0.08; Table 4). There were no correlations with other leukocyte subsets present in the decidua, however when samples from women with PTLI were included, there were highly significant positive correlations between CCL4, CCL5, CXCL1, CXCL8 and CXCL10 and neutrophil numbers (p<0.05, Table 4). Protein expression of CCL4, CCL5, CXCL1 and CXCL10 correlated with total leukocyte presence in the decidua (p<0.05), while CCL4 and CXCL8 protein expression correlated with neutrophil numbers (p<0.05, Table 4). There were no significant correlations between the expression levels of any of the chemokines and numbers of macrophages in the decidua.

**Table 4:** Correlations between choriodecidual chemokine mRNA/protein expression and leukocyte subtypes present in decidua.

	CD45		N Elastase		CD68			CD45		N Elastase		CD68	
Chemokine mRNA	r	*p*	r	*p*	r	*p*	Chemokine protein	r	*p*	r	*p*	r	*p*
**CCL2**	**0.15**	*0.49*	**0.36**	*0.049* *	**0.25**	*0.25*	**CCL2**	**0.15**	0.44	**0.05**	0.79	**0.28**	0.16
**CCL4**	**0.23**	*0.29*	**0.49**	*0.005* **	**0.38**	*0.08*	**CCL4**	**0.55**	0.0026**	**0.39**	0.04*	**0.21**	0.29
**CCL5**	**0.21**	*0.32*	**0.43**	*0.01* **	**0.48**	*0.027* *	**CCL5**	**0.49**	0.008**	**0.31**	0.11	**0.05**	0.79
**CCL8**	**−0.03**	*0.88*	**0.28**	*0.13*	**0.38**	*0.08*	**CCL8**	**0.26**	0.24	**0.19**	0.38	**−0.18**	0.4
**CXCL1**	**0.13**	*0.56*	**0.54**	*0.002* **	**0.40**	*0.06*	**CXCL1**	**0.46**	0.03*	**−0.03**	0.91	**0.16**	0.47
**CXCL8**	**0.024**	*0.91*	**0.48**	*0.007* **	**0.27**	*0.23*	**CXCL8**	**0.33**	0.08	**0.6**	0.0008***	**0.22**	0.28
**CXCL10**	**0.05**	*0.79*	**0.16**	*0.59*	**0.43**	*0.047* *	**CXCL10**	**0.48**	0.02*	**−0.24**	0.27	**0.04**	0.84

Spearman’s correlation.

* p<0.05,

** p<0.01,

** p<0.001.

### Immunohistochemistry

**Figure 4:**
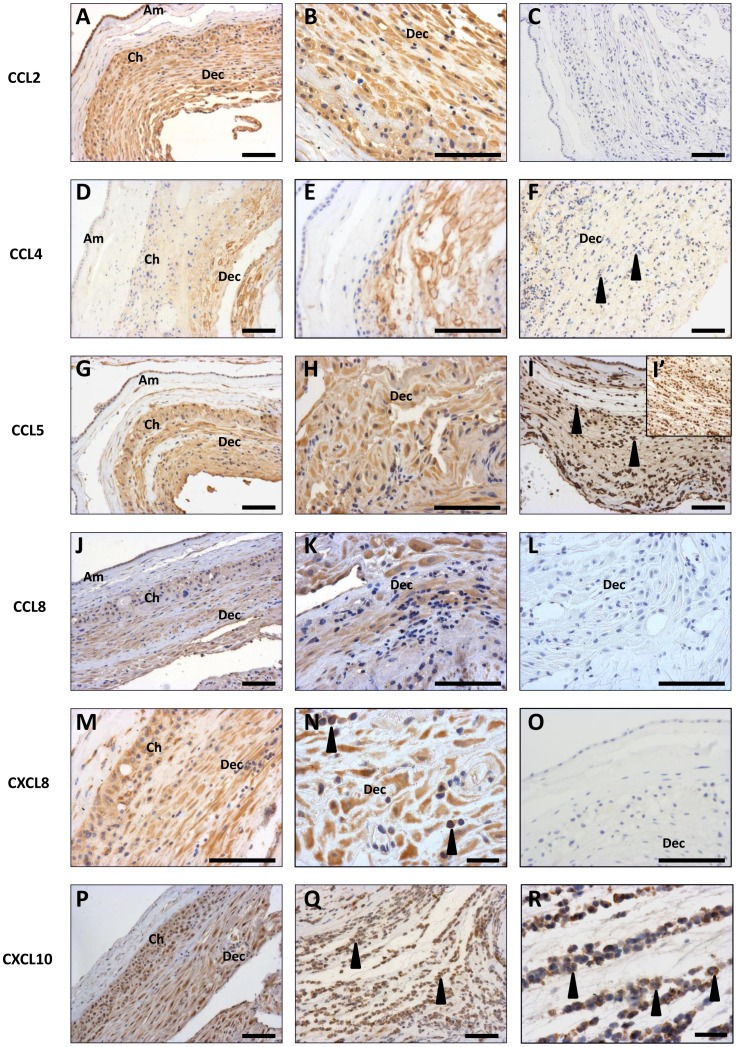
Localisation of chemokine proteins in fetal membranes. Representative images taken at original magnification of x10 or x20 for: (A–C) CCL2, (D–F) CCL4, (G–I) CCL5, (J–L) CCL8, (M–O) CXCL8, (P–R) CXCL10. Representative negative controls shown for CCl2 (C), CCL8 (L) and CXCL8 (O). Inset in Fig. I (I’) is a serial section immunostained for neutrophil-elastase (neutrophil marker) as decrsibed in [12], demonstrating the similarity between CCL5/CXCL10 immunostaining and neutrophil abundance in PTLI samples. Am = amnion, Ch = chorion, Dec = decidua. Arrow heads indicate cells with the morphologically appearance of leukocytes. All scale bars are 100 µm, except Figs N and R where they are 25 µm.

Immunohistochemistry was performed for the 6 chemokines confirmed to be upregulated in TL and/or PTL, to confirm production by the decidua, as the previous mRNA and protein expression analyses were performed on choriodecidua homogenates. All chemokines were localised to the decidua, with staining present in decidual stromal cells (Fig. 4). Most chemokines were also present in the chorion trophoblast layer, with the exception of CCL4 (Fig. 4D–F). Leukocytes are a potential source of chemokines, and positive staining for CCL2, CCL5, CXCL8 and CXCL10 was detected in cells within the decidual stromal with the morphological appearance of immune cells. Staining for CCL4 and CCL8 was confined to stromal cells within the decidual layer (Fig. 4F and K). In cases of PTLI, CCL5 and CXCL10 were strongly localised to the highly abundant leukocytes, identified as such from prior analyses of neutrophil infiltrate and their polymorphonuclear appearance (Fig. 4I and R).

### Chemokines in Maternal Plasma during Term and Preterm Labour

**Figure 5:**
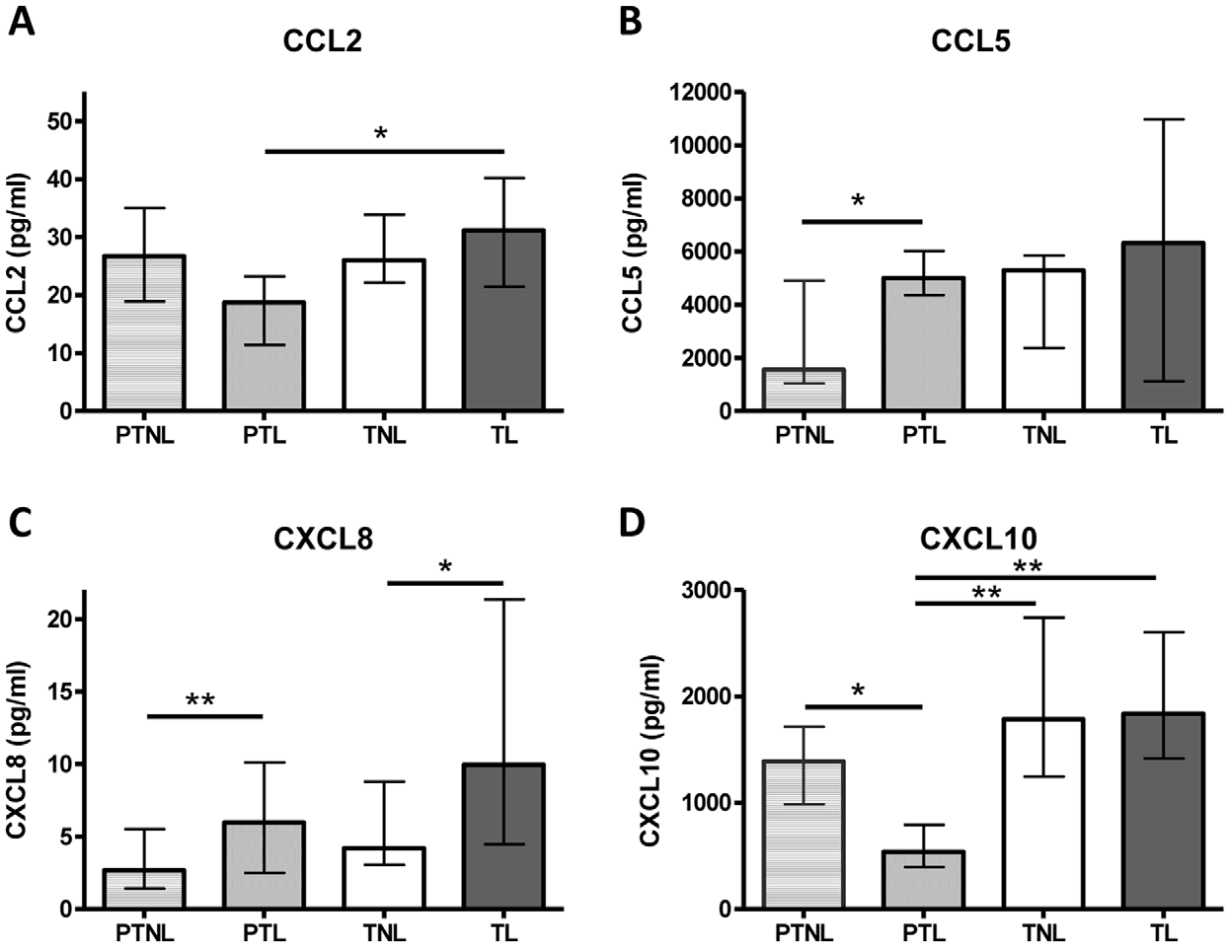
Chemokine concentrations in plasma from women in preterm and term labour. (A) CCL2, (B) CCL5, (C) CXCL8, (D) CXCL10. Individual replicates and the median are shown, n = 6–10/study group. PTNL = Preterm not in labour, PTL = Preterm labour, TNL = Term not in labour, TL = Term labour. Data are median and interquartile range, *p<0.05, **p<0.01 (Kruskall-Wallis with Dunn’s post hoc test).

To determine whether the chemokines of interest were elevated in maternal plasma during labour, Bioplex assays were performed on plasma samples from women in TL and PTL, compared to women not in labour at 28 weeks gestation (PTNL) and at term (TNL). All chemokines were detectable in maternal plasma. The only chemokine significantly altered by labour was CXCL8, which was present at higher concentrations in PTL and TL, compared to the relevant non-labouring groups (p<0.01, Fig. 5). CCL5 was significantly higher in PTL than PTNL (p = 0.05), but was unaltered by TL. CCL2 and CXCL10 concentrations were significantly lower in PTL than TL (p<0.05), with a similar trend for CXCL8 (p = 0.07).

### Regulation of Chemokine Expression by Betamethasone

**Figure 6:**
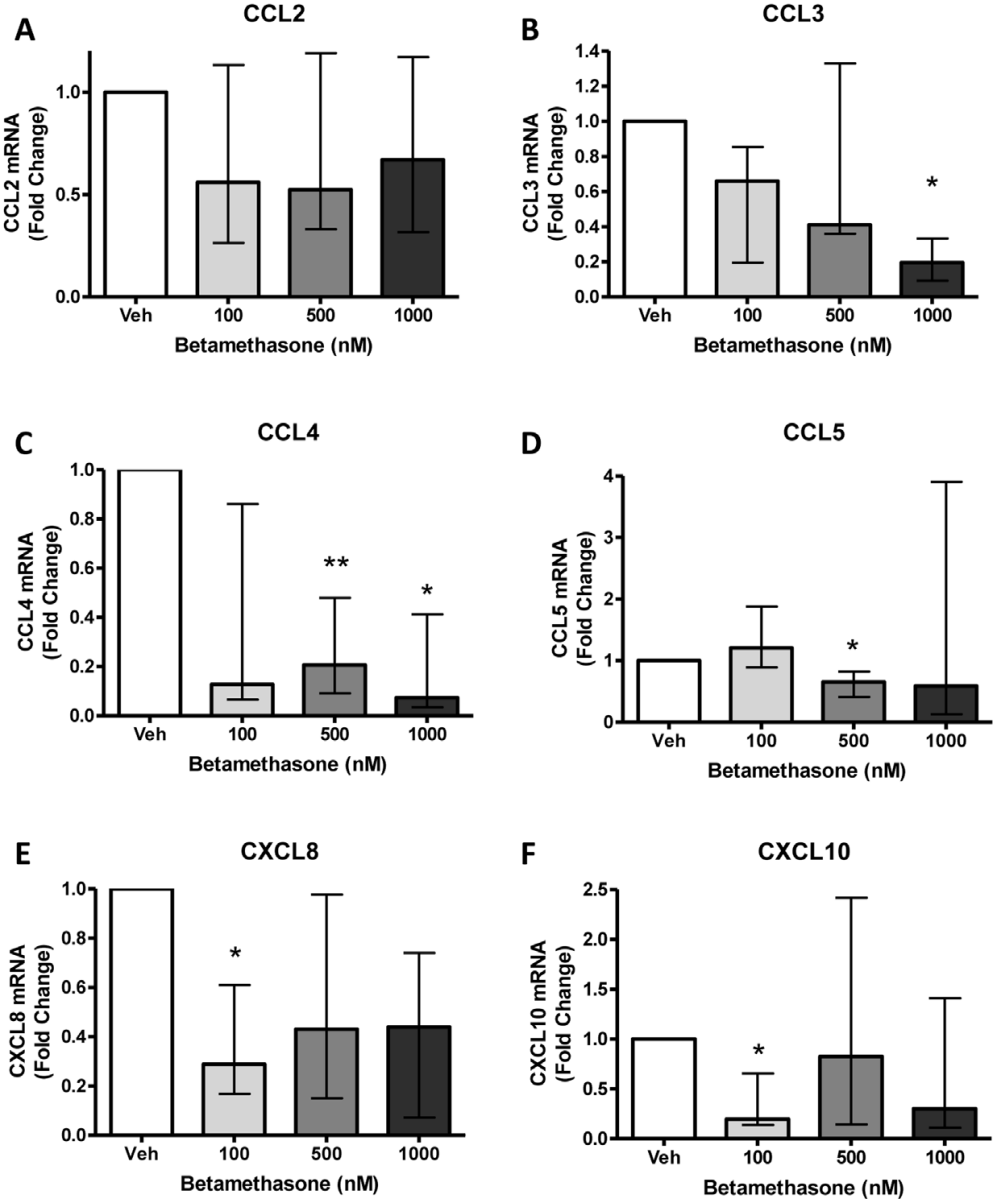
Effect of betamethasone on chemokine mRNA expression by choriodecidual explants. (A) CCL2, (B) CCL3, (C) CCL4, (D) CCL5, (E) CXCL8, (F) CXCL10. Data are fold change from vehicle control, n = 7 per treatment. Data are median and interquartile range, *p<0.05, **p<0.01 compared to vehicle control (Wilcoxon Signed Rank test).

All women delivering in the PTL and PTLI groups received at least one dose of betamethasone (12 mg/intramuscularly) prior to delivery, to promote fetal lung maturation. The interval between administration and delivery was generally 24–48 hours, although 3 women in the PTL group delivered within 12 hours. To investigate a potential suppression of decidual chemokines by betamethasone administered to women in PTL for fetal lung maturation, choriodecidual explants were treated with betamethasone for 24 hours and chemokine mRNA expression assessed. CCL3, CCL4, CCL5, CXCL8 and CXCL10 were significantly downregulated by betamethasone (p<0.05, Fig. 6). CCL2 was unaltered by betamethasone treatment.

## Discussion

There is growing awareness of the complex cellular and molecular changes in intrauterine tissues in preparation for and during labour, many of which focus around inflammatory pathways. However, there is relatively little known about inflammatory processes in the decidua. The current study aimed to address this gap of knowledge, and specifically to identify chemotactic factors responsible for instigating and regulating the influx of maternal immune cells into the decidua during labour, both at term and preterm. We detected a global elevation in decidual chemokine expression with labour, and identified 5 chemokines (CCL2, CCL4, CCL5, CXCL8 and CXCL10) which were upregulated at both the mRNA and protein level in TL compared to TNL, whilst CCL8 was elevated in PTL. These are predominantly monocyte/macrophage chemoattractants, which is consistent with the rise in macrophage numbers in the decidua during labour [12]. Macrophages have been hypothesized to participate in the labour process through the local activation and amplification of inflammatory responses [9], [14], [29] and play a similar role in tissue remodelling events in the cervix and myometrium [13], [14]. The similarities in inflammatory processes in decidua and other uterine tissues support the hypothesis of parallel orchestrated events in gestational tissues during labour.

Two of the chemokines identified (CCL2: formally known as MCP-1, monocyte chemoattractant protein-1 and CXCL8: interleukin-8, IL-8) have been widely studied in the context of labour and are expressed in gestational tissues (myometrium, cervix, decidua) [18], [20], [25], [30], [31], [32], [33], [34]. Whilst the upregulation of CCL2 during labour was unsurprising as it coincides with elevated macrophage numbers in the decidua, high expression of neutrophil chemoattractant CXCL8 in tissues largely lacking neutrophil infiltration was unexpected. However, both CCL2 and CXCL8 have additional roles in mediating T cell and dendritic cell chemotaxis which may contribute to the specialised decidual immunological environment. CXCL10 (also known as interferon gamma-induced protein 10, IP-10) was also identified as a potential regulator of leukocyte trafficking. Unlike most CXC chemokines, CXCL10 exhibits chemotactic activity to monocytes/macrophages, T cells, NK cells, and dendritic cells, rather than neutrophils [35]. CXCL10 has been attributed roles decidual NK cell recruitment and trophoblast migration in early pregnancy, but has not been identified in the late gestation decidua [36], [37]. Its expression strongly correlated with macrophage numbers reinforcing a role in normal term labour.

CCL4 (formally MIP-1β, macrophage chemotactic protein-1β) and CCL5 (RANTES) chemoattract monocytes, with additional activity towards NK cells and T cells respectively. Although both have been identified in the non-pregnant and early pregnancy uterus [38], [39], [40], [41], this is the first report of their upregulation in the decidua at TL and PTL. Their expression correlated with macrophage abundance, but also with neutrophil abundance suggesting this immune cell subtype is a major source of the chemokine. This was particularly apparent using immunostaining for CCL5 in fetal membranes from women with PTLI. Although they bind to a number of receptors, CCL4, CCL5 and CCL8 interact with CCR5, a receptor present on T cells and macrophages which has been the target of much study due to its involvement in HIV infection of CD4^+^ cells [42]. The redundant nature of the chemokine superfamily may potentially be exploited by blockade of a single receptor to inhibit a number of chemokine ligands. Whether inhibition of a subset of chemokines would be sufficient to reduce decidual immune cell trafficking and suppress inflammation during PTL remains unknown. However, individual chemokine receptor knockout mice do not exhibit a dysfunctional labour phenotype [43], suggesting targeting multiple pathways may be necessary.

In contrast to our previous reports of similar patterns of macrophage infiltration in TL and idiopathic PTL, very few of the changes in chemokine expression observed in TL were detected in PTL. The global chemokine profiles in pooled samples were similar; however, the majority of these changes were not validated by Q-PCR. This is due to considerable variability in chemokine expression levels between individual PTL samples, with outliers skewing the array data. This is in keeping the probable heterogeneous nature of the idiopathic PTL group, with differing aetiologies. Larger sample sizes are likely to be needed to reduce the variability and potentially examine distinct subgroups within the study group. There may be distinct activation pathways in preterm and term labour. Higher numbers of CD56+ NK cells and T cells in the decidua in PTL compared to TL [12], in concert with elevated expression of CCL8 (NK cell and T cell chemoattractant [44]) in the decidua during PTL support this theory. Alternatively, the differences in chemokine expression between PTL and TL may reflect the differences in gestational age of the samples. Whilst we are unaware of any data regarding changes in leukocyte/chemokine expression in the decidua on a week-to-week basis between 24 weeks and term, there may be preparatory processes for labour with advancing gestation. It is an unavoidable limitation of the current study that we were not able to decipher gestational age effects by comparing preterm decidua from women who had laboured and those who had not. However, it is not possible to obtain decidua from normal pregnancies that are preterm but not in labour, without the considerable confounders of pregnancy complications, such as pre-eclampsia and severe fetal growth restriction. Both of these conditions are associated with inflammation and/or abnormalities in the decidual environment [45], so would be inappropriate controls to compare to PTL due to significant confounding effects. It is also noteworthy than preparatory events for labour may have commenced in the pregnancies in the TNL group (which were obtained at >39 weeks gestation), as recently described for NFκB activation in the amnion [46]. Thus the stimulation of inflammatory responses (both in terms of leukocyte numbers and chemokine expression) in TL and PTL may be even greater than observed in this study.

Another further explanation for the lower choriodecidual chemokine expression in PTL is suppression of chemokine expression by betamethasone. All women delivering in the PTL and PTLI groups received betamethasone to promote fetal lung maturation. Chemokines are classically inhibited by glucocorticoids [47], [48], [49], and the decidua is a target tissue for glucocorticoids [50]. Thus choriodecidual explants were treated in vitro with clinically relevant concentrations of the glucocorticoid. The general suppressive action observed supports the theory that antenatal corticosteroid administration contributes to the lower levels of decidual chemokine expression in women in PTL compared to term labour. This is an unavoidable limitation in studies of PTL and thus studies using in vitro and animal models are likely to be necessary in conjunction with clinical studies such as these. It is noteworthy that the patients in the PTL study group also received tocolytics (Atosiban or Nifedipine); these are not envisaged to alter decidua chemokine production, but this has not been formally assessed.

Considerable differences were detected between PTL and PTLI in global chemokine expression patterns, with fewer CCL chemokines and a preponderance of CXC chemokines in PTLI. This is consistent with distinct decidual leukocyte subtypes between the two classes of PTL, with PTLI associated primarily with neutrophil infiltration, with no increase in macrophage infiltration [12]. These patterns were maintained in the QPCR and Bioplex assays, with most chemokines expressed at greatest levels in PTLI. Notably, protein levels for CCL4 and CXCL8 were >20-fold and >40-fold higher respectively in choriodecidua from women with infection-associated PTL than idiopathic PTL and both strongly correlated with neutrophil abundance. Not all of the chemokines elevated in PTLI are neutrophil chemoattractants, however neutrophils infiltrating the decidua express a plethora of chemokines and pro-inflammatory cytokines capable of activating local cells to recruit other cell types to combat the infection [51]. This was particularly evident in immunohistochemical studies of CCL5 and CXCL10, which were strongly expressed by infiltrating neutrophils. The loss of normal tissue architecture in PTLI explains the downregulation of the CCL chemokines, which are predominantly expressed by decidual stromal cells.

The combined approach of mRNA and protein analyses enabled refinement of the list of candidate chemokines mediating decidual leukocyte infiltration during labour from those originally identified by gene array. Changes in mRNA do not necessarily reflect changes at the protein level, as was seen for several chemokines (CCL3, CXCL1 and CXCL9). Those upregulated at the protein level are the strongest candidates for mediating monocyte/macrophage infiltration during term labour, however an involvement of other chemokines not validated cannot be excluded. For both the mRNA and protein analyses, choriodecidual homogenates were used, as it was not possible to isolate pure decidua; thus it was important to verify a decidual source of the chemokines. Using immunohistochemistry, all 6 candidate chemokines localised to decidual stromal cells, although the chorion layer also frequently exhibited positive staining. This was not anticipated as there are very few leukocytes present in the chorion layer compared to the decidua [12], , highlighting the fact that immunoreactive protein is not necessarily secreted. Notwithstanding this limitation, these studies do verify that decidua cells have the capacity to produce the chemokines of interest and thus could play an integral paracrine role in leucocyte recruitment and activation. The additional immunostaining for CXCL8 and CCL5 by cells morphologically identified as leukocytes in the decidua implies further amplification of leukocyte recruitment. This could be confirmed by dual immunofluoresence staining with leukocyte subtype markers in future studies.

Chemokine levels in maternal blood were also examined in women in PTL and TL. There are marked alterations in maternal peripheral leukocytes during labour at term and preterm, including elevated numbers of monocytes and neutrophils, and increased activation of neutrophils [23]. We report elevated circulating concentrations of CXCL8 in PTL and TL, and higher CCL5 in PTL compared to their respective non-labouring controls. These chemokines could act to prime maternal peripheral leukocyte activation and facilitate their entry into gestational tissues. The source of these chemokines is unknown, however, systemic changes that reflect inflammatory processes in the intrauterine environment is of clinical interest as potential screening tools for PTL. The utility of these changes in diagnosing true versus threatened PTL is worthy of further investigation; indeed a recent study identified CCL5 as a potential predictive marker for PTL in combination with serum IL-10 and cervical length [53]. The differences in CCL5 levels between PTL and TL may reflect alterations in expression of this chemokine with advancing gestation; this could be studied in a detailed study of longitudinal maternal plasma chemokine level across pregnancy.

To reinforce causal relationships between choriodecidual chemokine expression and leukocyte infiltration, correlation analyses were performed assessing samples from all study groups together. Cases of PTLI were excluded from most analyses as the cellular morphology of the decidua is often heavily disrupted by the substantial neutrophil infiltrate, thus the contribution of the decidual cells is impossible to assess. At the mRNA level there were positive correlations between macrophage numbers and several of the chemokines studied (CCL4, CCL5, CCL8 and CXCL10), thus it is tempting to speculate these chemokines contribute to their recruitment. However, these correlations may also represent expression of chemokines by resident macrophages. Disappointingly, these relationships were not observed at the protein level, although these chemokines did correlate with the number of total CD45^+^ leukocytes, the majority of which are macrophages. To investigate chemokines participating in the neutrophil influx further correlation analyses were performed including the PTLI group. Very strong correlations were detected between neutrophil numbers and most chemokines, particularly CCL4 and CXCL8, which were maintained at the protein level. The distinct chemokine profiles and relationships with leukocyte subtypes in PTL and PTLI reinforce distinct aetiologies in these two study groups.

In summary, this study has identified a number of chemokines that may be responsible for the decidual inflammatory infiltrate in both PTL and term labour. The main chemokines confirmed to be upregulated at the mRNA and protein level in labour are CCL2, CCL4, CCL5, CCL8, CXCL8 and CXCL10. Of these, CCL2, CCL4, CCL5, CCL8 and CXCL10 are the most likely candidates for attracting macrophages into the decidua during labour, with CXCL8 fulfilling an as yet unidentified role in the normal labour process. These studies support the hypothesis that decidual- and leukocyte-derived chemokines act in concert to regulate leukocyte recruitment to the decidua in TL and PTL. Our parallel studies demonstrating that leukocyte infiltration of the decidua precedes labour in rat pregnancies suggests a causal role in the labour process, and therefore chemokines regulating leukocyte trafficking may represent a potential therapeutic target for the prevention of PTL.

## Supporting Information

Table S1 **Inflammatory genes differentially expressed in choriodecidua with labour.** Term labour (TL), idiopathic preterm labour (PTL) and infection associated preterm labour (PTLI). Data were normalised for the expression of RPL13a and presented as fold change from expression levels in choriodecidua from term pregnancies in the absence of labour (TNL). *U = upregulated in labour, but not expressed in TNL, D = downregulated in labour, but not expressed in TNL, − = not expressed.*
(DOCX)
