# Supplementary material for: Identification of Chemokines Associated with the Recruitment of Decidual Leukocytes in Human Labour: Potential Novel Targets for Preterm Labour
Source: PLoS One. 2013 Feb 22;8(2):e56946. doi: 10.1371/journal.pone.0056946 (PMC3579936; doi:10.1371/journal.pone.0056946)
Supplement: Table S1 — Inflammatory genes differentially expressed in choriodecidua with labour. Term labour (TL), idiopathic preterm labour (PTL) and infection associated preterm labour (PTLI). Data were normalised for the expression of RPL13a and presented as fold change from expression levels in choriodecidua from term pregnancies in the absence of labour (TNL). U = upregulated in labour, but not expressed in TNL, D = downregulated in labour, but not expressed in TNL, − = not expressed. (DOCX) [file pone.0056946.s001.docx]

**Table S1.** **Inflammatory genes differentially expressed in choriodecidua with labour.** Term labour (TL), idiopathic preterm labour (PTL) and infection associated preterm labour (PTLI). Data were normalised for the expression of RPL13a and presented as fold change from expression levels in choriodecidua from term pregnancies in the absence of labour (TNL)*.* U = upregulated in labour, but not expressed in TNL, D = downregulated in labour, but not expressed in TNL, - = not expressed.

| **Gene** | **TL vs. TNL**  **(fold change)** | **PTL vs. TNL**  **(fold change)** | **PTLI vs. TNL**  **(fold change)** |
| --- | --- | --- | --- |
| CSF3 | 197.33 | 164.74 | 125.88 |
| CXCL2 | 119.80 | 42.64 | 24.86 |
| CCL11 | 45.08 | 9.41 | D |
| CXCL11 | 44.46 | 17.93 | 7.04 |
| SCYE1 | 42.06 | 2.59 | D |
| CCRL1 | 38.17 | 14.97 | D |
| CXCL1 | 35.13 | 27.17 | 59.13 |
| IL8 | 29.95 | 26.98 | 144.59 |
| CCL8 | 25.89 | 7.33 | 1.84 |
| C5 | 25.71 | 4.90 | -1.04 |
| CCRL2 | 23.66 | 10.66 | -35.61 |
| CMTM2 | 23.66 | 6.47 | -4.42 |
| IL1A | 21.03 | 17.56 | 66.53 |
| CXCL5 | 18.44 | 16.73 | 58.32 |
| CCR2 | 17.56 | 3.49 | -2.37 |
| CXCL10 | 17.44 | 14.87 | 10.03 |
| CCL18 | 16.27 | 26.98 | -1.54 |
| CCL17 | 13.88 | 2.78 | 2.56 |
| CCL4 | 13.22 | 7.49 | -16.16 |
| BLR1 | 12.68 | 3.06 | 1.98 |
| CCL13 | 12.51 | D | -1.49 |
| GPR31 | 12.25 | 1.22 | 1.75 |
| CCR5 | 11.75 | 18.05 | 13.14 |
| BDNF | 11.19 | 1.66 | D |
| IL8RA | 10.81 | 9.41 | D |
| CCL16 | 9.88 | -14.38 | D |
| TLR2 | 9.48 | 17.32 | 18.45 |
| CCBP2 | 9.28 | 3.82 | -336.42 |
| CCR7 | 9.03 | 5.91 | -1.91 |
| CXCR4 | 7.86 | 8.54 | 17.22 |
| C5AR1 | 7.49 | 4.24 | -767.56 |
| LTB4R | 6.79 | 2.88 | -123.14 |
| SLIT2 | 6.25 | 2.35 | 2.52 |
| CXCL9 | 5.29 | 3.93 | 2.65 |
| CCL1 | 5.19 | 1.43 | -10.88 |
| CCR10 | 5.15 | 1.56 | 1.33 |
| **Gene** | **TL vs. TNL**  **(fold change)** | **PTL vs. TNL**  **(fold change)** | **PTLI vs. TNL**  **(fold change)** |
| TNF | 4.51 | 3.13 | 59.54 |
| CCL5 | 4.42 | 2.84 | 1.32 |
| HIF1A | 4.01 | 4.33 | -253.20 |
| CCL2 | 3.88 | 2.96 | 11.52 |
| XCL1 | 3.80 | 1.12 | -18.95 |
| CCR6 | 3.74 | 1.14 | 1.44 |
| VHL | 3.57 | 2.66 | -9.67 |
| CX3CL1 | 3.47 | 2.49 | -1.33 |
| GPR81 | 3.37 | -1.74 | -2.45 |
| CYFIP2 | 3.26 | 1.14 | 1.18 |
| CCR1 | 3.19 | 1.61 | 5.23 |
| CCL19 | 2.74 | -1.87 | -2.26 |
| TNFSF14 | 2.67 | 1.91 | 15.52 |
| TLR4 | 2.61 | 4.01 | 4.91 |
| NFKB1 | 2.45 | 2.57 | -408.48 |
| CCL3 | 2.39 | 1.72 | 34.92 |
| CXCL13 | 2.27 | 1.58 | 2.20 |
| SDF2 | 2.26 | 1.65 | 1.26 |
| TREM1 | 2.09 | 1.67 | 6.17 |
| MYD88 | 2.01 | 1.70 | 4.40 |
| AGTRL1 | U | U | U |
| CCL15 | U | U | - |
| CCL7 | U | U | U |
| CCR3 | U | U | U |
| CCR4 | U | U | U |
| CCR8 | U | U | U |
| CX3CR1 | U | U | - |
| CXCL3 | U | U | U |
| CXCL6 | U | U | U |
| GDF5 | U | U | - |
| IL13 | U | U | - |
| IL4 | U | U | - |
| TCP10 | U | U | - |
| MMP2 | 1.99 | 2.26 | 1.48 |
| IL18 | 1.71 | 2.59 | 1.70 |
| CMKLR1 | 1.46 | 1.72 | -1.66 |
| CXCL12 | 1.41 | 1.92 | 2.11 |
| CKLF | 1.36 | 1.05 | 1.87 |
| TNFRSF1A | 1.27 | -1.40 | -1.40 |
| CMTM3 | 1.25 | -1.10 | -1.43 |
| CXCR6 | 1.17 | 1.10 | -1.98 |
| ECGF1 | 1.05 | 1.04 | -1.13 |
| CXCR3 | -1.05 | 1.05 | -4.61 |
| **Gene** | **TL vs. TNL**  **(fold change)** | **PTL vs. TNL**  **(fold change)** | **PTLI vs. TNL**  **(fold change)** |
| IL16 | -1.07 | 1.02 | 1.02 |
| CMTM4 | -1.08 | -3.29 | -3.93 |
| CMTM1 | -1.29 | -2.11 | -1.52 |
| XCR1 | -1.63 | -7.09 | D |
| MMP7 | -4.27 | -1.28 | -3.26 |
